# Genome-wide assessment of imprinted expression in human cells

**DOI:** 10.1186/gb-2011-12-3-r25

**Published:** 2011-03-21

**Authors:** Lisanne Morcos, Bing Ge, Vonda Koka, Kevin CL Lam, Dmitry K Pokholok, Kevin L Gunderson, Alexandre Montpetit, Dominique J Verlaan, Tomi Pastinen

**Affiliations:** 1 McGill University and Genome Quebec Innovation Centre, 740 Dr Penfield Avenue, Montreal, Quebec, H3A 1A4, Canada; 2 Illumina Inc., 9885 Towne Centre Drive, San Diego, CA 92121, USA

## Abstract

**Background:**

Parent-of-origin-dependent expression of alleles, imprinting, has been suggested to impact a substantial proportion of mammalian genes. Its discovery requires allele-specific detection of expressed transcripts, but in some cases detected allelic expression bias has been interpreted as imprinting without demonstrating compatible transmission patterns and excluding heritable variation. Therefore, we utilized a genome-wide tool exploiting high density genotyping arrays in parallel measurements of genotypes in RNA and DNA to determine allelic expression across the transcriptome in lymphoblastoid cell lines (LCLs) and skin fibroblasts derived from families.

**Results:**

We were able to validate 43% of imprinted genes with previous demonstration of compatible transmission patterns in LCLs and fibroblasts. In contrast, we only validated 8% of genes suggested to be imprinted in the literature, but without clear evidence of parent-of-origin-determined expression. We also detected five novel imprinted genes and delineated regions of imprinted expression surrounding annotated imprinted genes. More subtle parent-of-origin-dependent expression, or partial imprinting, could be verified in four genes. Despite higher prevalence of monoallelic expression, immortalized LCLs showed consistent imprinting in fewer loci than primary cells. Random monoallelic expression has previously been observed in LCLs and we show that random monoallelic expression in LCLs can be partly explained by aberrant methylation in the genome.

**Conclusions:**

Our results indicate that widespread parent-of-origin-dependent expression observed recently in rodents is unlikely to be captured by assessment of human cells derived from adult tissues where genome-wide assessment of both primary and immortalized cells yields few new imprinted loci.

## Background

Most mammalian autosomal genes are thought to be expressed co-dominantly from the two parental chromosomes. At some loci, the allele inherited from one parent is suppressed through epigenetic mechanisms. This monoallelic expression, referred to as imprinting, leads to genetic vulnerability that can contribute to rare monogenic syndromes, such as Angelman and Prader-Willi syndromes [1]. Recent evidence suggests that common disease, such as basal-cell carcinoma and type 2 diabetes, can also be impacted by parent-of-origin-specific allelic variants [2]. Classical imprinting of a region is the result of expression of only one parental allele, where the other allele is completely suppressed. However, a more subtle imprinting effect has been recently reported where both alleles are differently expressed and show this in a parent-of-origin-dependent manner. This deviation of typical imprinting is called partial imprinting [3].

Although there is no global explanation for the role of imprinting in mammalian development and physiology, a parental conflict over the distribution of resources to offspring theory has been hypothesized [4], and reviewed in [5]. When maternal and paternal input in the offspring is unequal, a differing evolutionary pressure is placed on the alleles inherited from one or the other parent, where the maternally derived allele acts to decrease maternal contribution to the fetus and the paternally derived allele acts to increase maternal contribution [4]. Imprinted genes have been shown to be very important in fetal, placental and brain development, postnatal growth, behavior and metabolism [6]. However, since not all imprinted genes are involved in development or growth and imprinting, they have likely evolved more than once [7].

The debate around theories of imprinting parallels the intense investigation of the mechanisms that maintain imprinting. Monoallelic expression can be achieved with mechanisms such as CpG island methylation, histone modifications, antisense transcript-associated silencing, as well as by long-range chromatin effects [8]. However, such allele-specific phenomena are not restricted to imprinted genes [9] and not all of these mechanisms can be found in every imprinted locus. Because of this, studies looking at individual attributes of chromatin structure without correlation to gene expression may not be efficient in uncovering imprinted genes [10].

Although there are several genomic parameters that seem to distinguish imprinted and non-imprinted genes (smaller introns, repeat sequences), which have been exploited in attempts to bioinformatically predict mammalian imprinted genes [11,12], these characteristics are not found in all imprinted genes. A feature of these predictions is the generation of a large number of potentially imprinted genes; for example, one study predicted 600 imprinted genes [13] while another predicted that there may be over 2,000 imprinted genes [14]. Yet, few of these bioinformatic predictions have been validated [15], leading many to believe that the numbers are largely inflated and that the number of imprinted genes yet to be identified is small [9]. More conservative estimates assume 100 to 200 imprinted genes in the human genome [16].

So far, direct observation of mammalian imprinting in living cells and tissues has been carried out most thoroughly in the mouse genome using RNA-seq [17,18]. These studies employed the gold standard for recognizing imprinting in mice using the non-equivalence of monoallelic expression in reciprocal matings of inbred strains but yielded widely different estimates of amounts of imprinted genes in mouse embryonic brain. Using three brain regions, up to 1,300 transcripts were reported as imprinted [18], whereas a single brain region studied for 5,000 genes observed only a handful of novel imprinted genes beyond the more than 100 validated earlier [17]. Criteria for calling imprinting allowed for partial and inconsistent parent-of-origin-dependent expression within transcripts and between individuals and along with shown tissue specificity [18] may, in part, explain the substantial discrepancy between the two studies. The reciprocal mating approach used with mice cannot be used with humans. Consequently, demonstration of imprinting requires family-based tissue samples as well as accurate methods to observe differential expression of parental alleles. An obvious limitation to human studies is the access to multiple tissue types where transmission patterns can be determined. This leads to some genes being reported as imprinted without clear demonstration of allelic expression (AE) bias [19] and/or parental bias [20-22]. Because of these limitations, it is unclear what the extent of imprinting is in humans. Currently, direct assessment of imprinting in human tissues has yielded approximately 80 genes with varying degrees of evidence for imprinting [23] and an up to date catalogue is kept at the Catalogue of Parent of Origin Effects [24]. Some of the imprinted genes have been found to be tissue- or developmental stage-specific [7]. Given the limitations in sampling as well as measuring differential expression of parental alleles comprehensively, it is commonly assumed that the number could be significantly higher.

In addition to imprinting, random monoallelic expression (RME) has been reported as a source of sequence-independent AE [25]. When RME occurs at a given locus, a range of expression can follow such that some cells express only the maternal allele, some cells express only the paternal allele and some cells express a combination of the two. This class of genes has been previously reported in the odorant receptor genes as well as genes encoding immunoglobulins, T-cell receptors, interleukins, and natural killer cell receptors [26-30]. Historically, RME was linked to a subset of genes involved in the immune or nervous system. However, Gimelbrant *et al*. [25] assessed 3,939 genes in multiple clonal lymphoblast cell lines (LCLs) and found that roughly 10% were monoallelically expressed and observed a large diversity in RME genes. In their study, different cell clones derived from the same individual showed biallelic behavior at most loci. Other studies have established links between allele-specific DNA methylation and RME [31]. In an earlier study of ours, we observed an excess of high-magnitude AE in immortalized lymphoblasts (LCL) compared to primary cells (osteoblasts and fibroblasts) and this correlated with the estimated levels of clonality [32]. It has been hypothesized that aberrant methylation induced by lymphoblast immortalization, prolonged cell culture or multiple passages may be a possible mechanism for the observed AE [33]. In this study, we utilize a genome-wide method [32] to determine strongly biased AE in the transcriptome using family-based cell panels from two cell types (lymphoblasts and primary fibroblasts). Using this method, we aim to uncover imprinting in the human genome by determining parent-of-origin transmission in multiple pedigrees as well as excluding heritable variants that cause monoallelic expression through population-based data obtained from these same samples. To globally assess the relationship between methylation and RME, we perturbed the methylation state in lymphoblasts using 5-azadeoxycytidine (AZA), a drug that causes hemi-demethylation, and monitored changes in AE upon demethylation. The density of measurements, inclusion of family- and population-based AE from two cell types along with an investigation of methylation impact on differential AE provides the most comprehensive survey of epigenetic *cis-*regulatory variation in the human genome to date.

## Results

### Validated imprinting in lymphoblast cell lines and fibroblasts

First, we assessed the level of evidence for non-overlapping genes suggested to be imprinted (Catalogue of Parent of Origin Effects [24]), specifically looking for demonstration of monoallelic expression with parent-of-origin-specific transmission in at least one pedigree. For genes with consistent parent-of-origin transmission, our search yielded a total of 44 imprinted genes. We were able to assess 73% of the confirmed imprinted genes (32 of 44) in either lymphoblasts or fibroblasts (Table 1; Table S1 in Additional file 1), as 12 loci were uninformative in our analysis (Table S2 in Additional file 1). The degree of allelic bias was extracted from the Illumina 1M AE assay [GEO:GSE26286] essentially as previously described [32].

**Table 1:** Validated imprinted genes in the human genome

Location	Gene	Transcript	Human	Mouse	Expressed allele	LCL	FB
6q24	*PLAGL1*^a^	NM_001080952	I	I	P	No	Yes
7q21	*SGCE*	NM_001099401	I	I	P	Yes	No
7q21	*PEG10*	NM_015068	I	I	P	NA	Yes
7q32	*CPA4*	NM_016352	I	NR	M	No	Yes
7q32	*MEST*	NM_177524	I	I	P	No	Yes
7q32	*COPG2*	NM_012133	CD	I	P	No	Yes
7q32	*KLF14*	NM_138693	I	I	M	NA	Yes
11p15	*H19*	NR_002196	I	I	M	No	Yes
11p15	*KCNQ1*	NM_000218	I	I	M	Yes	NA
14q32	*MEG3*	NR_002766	I	I	M	No	Yes
15q11	*MKRN3*	NM_005664	I	I	P	NA	Yes
15q11	*MAGEL2*	NM_019066	I	I	P	NA	Yes
15q11	*NDN*	NM_002487	I	I	P	NA	Yes
15q11	*SNURF*	NM_005678	I	I	P	Yes	Yes
15q11	*IPW*	NR_023915	I	I	P	Yes	Yes
16p13	*ZNF597*	NM_152457	I	NR	M	Yes	Yes
19q13	*ZNF331*	NM_001079906	I	NR	P	Yes	Yes
19q13	*ZIM2*	NM_015363	I	I	P	No	Yes
20q13	*GNAS/GNASAS*	NR_002785	I	I	M	Yes	Yes
20q13	*L3MBTL*	NM_032107	I	NR	P	Yes	Yes

^a^Only *PLAGL1 *isoform 1 is found expressed and imprinted in the fibroblasts; isoforms 1 and 2 are biallelically expressed in the LCLs. CD, conflicting evidence as defined by Morrison *et al*. [19]; FB, fibroblast cell lines; I, imprinted genes with previously observed parent-of-origin-dependent expression bias; LCL, lymphoblast cell lines; M, maternal; NA, not available (not expressed or non-informative in children); NR, not reported; P, paternal.

To validate the allelic expression calls from the Illumina 1M assay, we tested 15 SNPs from putative imprinted loci in 63 samples using a normalized Sanger sequencing-based validation assay [34]. One SNP gave discrepant genotyping calls and was excluded from the analysis, leaving 14 SNPs and 61 samples for comparison (Table S3 in Additional file 1). The analysis shows a concordant expression bias towards the expected allele in all cases with Pearson correlation coefficient of r = 0.9657 (Additional file 2).

**Figure 1:**
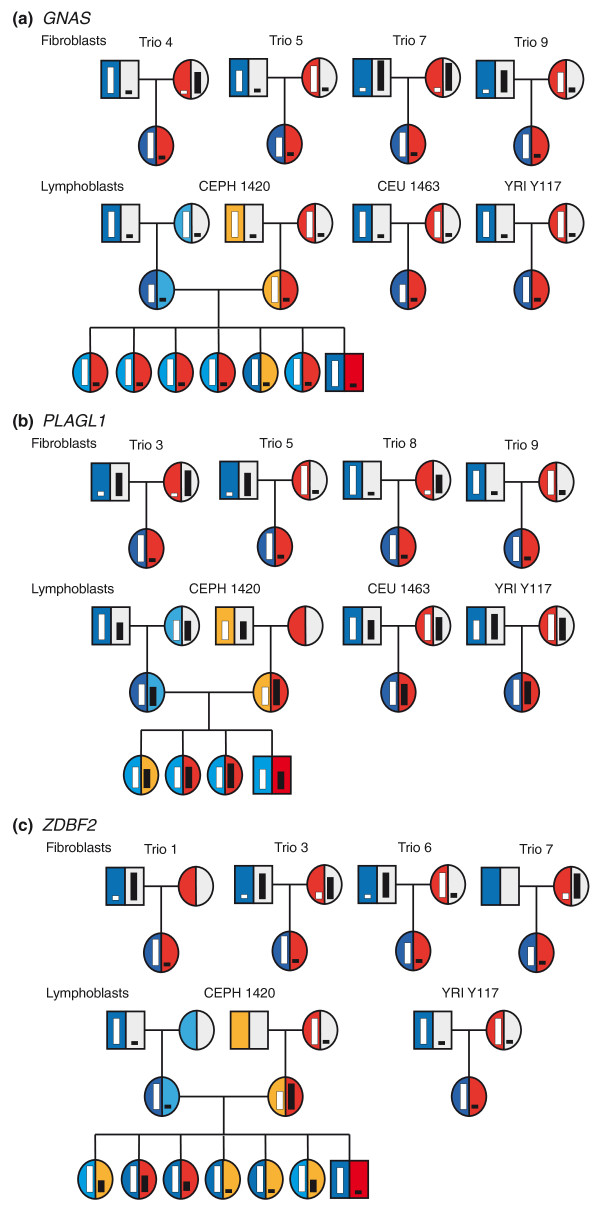
**Examples of imprinted genes in Human genome**. **(a) **Imprinted genes in both lymphoblasts and fibroblasts: *GNAS *is an example of an imprinted gene that has been previously described in the literature and has been confirmed in our study as well. **(b) **Imprinted genes in fibroblasts only: *PLAGL1 *is an example of tissue-specific imprinting (isoform 1). **(c) **Novel imprinted genes: *ZDBF2 *is an example of a novel imprinted gene. In each case, the figure shows all of the informative pedigrees. For the trios, the colors indicate the paternal allele (blue) and the maternal allele (red). For the three-generation pedigree the colors indicate which parental allele is inherited. The bars indicate which allele is overexpressed as well as the degree of overexpression.

The parent-of-origin-dependent transmission of allelic biases was confirmed in lymphoblasts using a three-generation pedigree of Caucasian origin (CEPH family 1420) [32] along with newly generated AE profiles in a Caucasian as well as a Yoruban parent-offspring trio. We also used nine independent parent-offspring fibroblast trios to confirm parental influence in AE. Of the known imprinted genes that were assessed, 37.5% (12 of 32) showed monoallelic expression and clear parental bias in either both tissues or in only one tissue if the other could not be assessed (Figure 1a and Table 1). Seven of these have been previously validated in LCLs by independent PCR-based AE measurements in a second pedigree (CEPH family 1444) [32]. An additional 22% (7 of 32) showed predominantly biallelic expression (average fold-difference between alleles < 2-fold) in one tissue with large magnitude AE and clear parental bias in the other tissue (Figure 1b and Table 1). For these 19 imprinted genes, the average increased expression of the overexpressed allele was 7.39-fold (2.94 to 11.84, 1 standard deviation (SD)). The remaining genes (13 of 32; 40%) all showed biallelic expression in all available measurements (Table S1 in Additional file 1). Overall, out of the 32 imprinted genes, we discovered that the AE observed for the genes *PRIM2*, *CPA4*, and *DLGAP2 *in LCLs was found to be associated with genotypes at local SNPs, consistent with heritable rather than imprinted allelic expression. Interestingly, the extreme AE observed for the *CPA4 *gene, although heritable in LCLs, is found to be consistent with imprinting in the fibroblasts.

Second, we looked for suggested imprinted genes (Catalogue of Parent of Origin Effects [24]), but with inconsistent parent-of-origin transmission data in the literature. Our search yielded 13 genes (marked 'PD/CD' in the tables), of which 69% (9 of 13) could be assessed. Only the gene *COPG2 *was validated for imprinting in the fibroblasts (Table 1) but was found to heritable in LCLs (data not shown). All of the remaining eight genes were found to be biallelic in lymphoblasts and/or fibroblasts (Table S1 in Additional file 1) and the AE observed for the genes *ZNF215 *and *GABRG3 *was found to be heritable in both cell types (data not shown).

### Novel imprinted genes and genomic regions

Using AE patterns observed for validated imprinted genes, which showed at least 2.9-fold difference in expression (-1 SD for confirmed imprinted genes), we sought evidence for imprinting among annotated genes and unannotated transcripts. We required that at least three consecutive SNPs showed an average deviation in excess of a 2.9-fold threshold and were measured in at least two children. Altogether, out of the 223,017 windows measured in at least two children, 1,253 fulfilled the criteria in the three-generation LCL pedigree, and of the 234,837 windows measured in the fibroblasts, a total of 549 were showing high AE. These candidate windows fell into 254 distinct loci in LCLs and into 110 loci in fibroblasts (Tables S5 and S6 in Additional file 3). Six of these loci in LCLs (spanning 8 genes) and 15 loci in fibroblasts (spanning 19 genes) had earlier literature evidence and were included in the assessment of known loci above. Our analysis revealed five imprinted RefSeq annotated genes not reported by other methods in humans (Table 2, Figure 1c). The genes *ZDBF2 *and *SGK2 *were found imprinted in LCLs, while the genes *NAT15*, *RTL1 *and *MEG8 *were found imprinted in fibroblasts. Three of these novel imprinted human genes had previously been identified in mice (*ZDBF2*, *RTL1*, *MEG8*) [35-37]. We note that in the fibroblasts, none of the regions overlapping RefSeq annotation and demonstrating potentially parent-of-origin-based transmission showed positive population mapping data (*n *= 15) whereas 36% (4 out of 11) for LCLs showed links with common variants in mapping data (Tables S5 and S6 in Additional file 3).

**Table 2:** Novel imprinted genes found in lymphoblasts and/or fibroblasts

				LCL		FB	
					
Location	Gene	Mouse	Expressed allele	Number of ITs	AE (average magnitude)	Number of ITs	AE (average magnitude)
2q33	*ZDBF2*	I	P	9	12.06	NA	NA
16p13	*NAT15*	NR	M	NA	NA	3	6.95
20q13	*SGK2*	NR	P	9	8.9	NA	NA
14q32	*RTL1*	I	P	NA	NA	4	12.34
14q32	*MEG8*	I	M	NA	NA	8	10.66

AE, allelic expression; FB, fibroblast cell lines; I, imprinted genes with previously observed parent-of-origin-dependent expression bias; IT, informative transmission; LCL, lymphoblast cell lines; M, maternal; NA, not available; NR, not reported; P, paternal.

**Figure 2:**
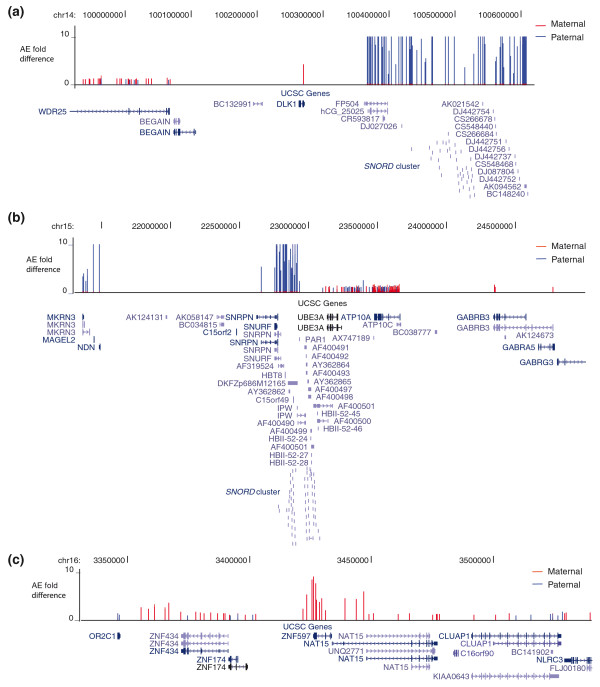
**Examples of imprinted genomic regions in fibroblasts**. **(a) **Paternally expressed imprinted region on chr14 covering numerous non-RefSeq genes found downstream of the paternally imprinted *DLK1 *gene (was not informative in our samples). This region has been previously identified in mice and sheep. **(b) **Extension of imprinting with paternal expression downstream of the *SNRPN*/*SNURF *loci encompassing multiple non-RefSeq genes. **(c) **Maternally expressed imprinted gene *ZNF597 *with upstream imprinted isoform-specific *NAT15*.

Since transcription was measured across the genome, we were able to observe potentially imprinted expression of ten unannotated intergenic regions (Table 3; Additional file 4). Four of these ten regions showed strong evidence for imprinting while the remaining six were found to be consistent with heritable AE. In some cases (*n *= 3) the imprinting regions spanned two to three genes and measured between 73,150 and 1,569,064 bases (Figure 2). We also commonly encountered imprinted transcription of SNPs outside the boundaries of annotated imprinted genes. For example, 10 of the 20 RefSeq genes showing strong evidence of imprinting continued this strong imprinted expression outside of the annotated gene boundary. Surprisingly, seven of these ten cases showed imprinted expression 5 kb away from the transcript, suggesting that they may represent independent transcriptional units or unannotated isoforms of the imprinted genes.

**Table 3:** Novel candidate imprinted intergenic regions in lymphoblasts and fibroblasts

Chromosome	Start	End	LCL AE (average magnitude)	FB AE (average magnitude)	Heritable AE
1	210509341	210524037	4.23	NI	Yes
1	72584492	72610078	NI	2.94	Yes
2	187422507	187893532	NI	2.68	No
7	26113744	26137739	4.36	NI	Yes
12	9514883	9649634	4.24	NI	Yes
14^a^	100425763	100608884	NI	8.58	No
15^b^	22786809	22902119	10.18	7.55	No
16	54019260	54035547	8.54	NI	Yes
16	3355563	3366918	NI	2.78	No
17	41604896	41620711	NI	5.87	Yes

^a^Downstream of *MEG3 *and *RTL1*. ^b^Within *SNRPN/SNURF *region. AE, allelic expression; FB, fibroblast; LCL, lymphoblast cell line; NI, not informative.

### Partial imprinting

We have previously shown that immortalized LCLs demonstrate an excess of monoallelic expression, putatively due to rare RME events detectable in these lines [32]. To avoid such biases, we looked for moderate magnitude AE (2- to 2.9 fold average difference among all informative heterozygotes) in loci where at least two of the children of the nine fibroblast trios were heterozygous to uncover partial imprinting. To avoid redundancy, we excluded AE at boundaries of classically imprinted regions (as defined in the above sections). Out of the 234,837 windows measured, we identified 46 loci that showed this degree of allelic bias. Of these, 30 could be determined to be consistent with heritable AE, mappable to local polymorphisms; in 80% of cases (24 of 30) the mapped polymorphism was transmitted in a Mendelian fashion (the remaining 6 were not informative for transmission of the putative regulatory variant). The remaining 16 RefSeq genes did not show association with common SNPs and were further investigated for change of relatively overexpressed haplotype with transmission (indicative of non-genetic effect) and parental bias in pedigrees. Four of the 16 showed strong evidence for partial imprinting, with the father's allele being preferentially expressed (*TRAPPC9*, *ADAM23*, *CHD7*, *TTPA*; Additional file 4).

### Mechanisms for random allelic expression

In order to assess the basis of extreme non-imprinted, non-heritable AE observed in lymphoblasts, three LCLs were treated with the demethylating agent AZA and were observed for changes in AE upon treatment. The three cell lines were selected based on our earlier data indicating high levels of clonality in these particular cell lines [32] based on extreme deviation from random X-inactivation. Using 5 μM AZA for 3 days, we observed a significant decrease in AE in 20% of loci that showed at least a two-fold difference in AE at baseline (defined as an allelic change of at least 1.25-fold, the 95th percentile of allelic fold change among untreated biological controls). Only one of the imprinted loci showed a change in AE upon treatment (*GNAS*). Similarly, loci where the AE could be mapped to common SNPs [32] were underrepresented: 23% (7 of 30) of AE traits affected by treatment mapped to SNPs (Table 4), whereas 35% (17 of 48) of loci without significant treatment effect on AE showed association with local SNPs (Table 5). These observations suggest that the demethylation alters the expression of randomly silenced genes in lymphoblasts. We studied this further by observing concordance of AE for identical-by-descent (IBD) siblings in a three-generation pedigree (CEPH 1420). We reasoned that if demethylation primarily affects random allelic silencing, then loci demonstrating treatment-specific effects would also more likely show random or IBD-independent AE since heritable or imprinted loci should demonstrate consistent AE. IBD siblings were considered concordant for AE if both had the same allele overexpressed and showed over 1.5-fold difference between alleles. They were considered discordant if one sibling showed 1.5-fold overexpression and the other sibling was either biallelic or overexpressed the other allele. The IBD sibling analysis showed discordant AE in 30% of transmissions for loci affected by treatment but only in 1% of loci not altered by treatment (*P*-value = 0.00308; Table 6). This suggests that RME, which is detectable in lymphoblasts due to their reduced mosaicism [32], may be partly explained by aberrant methylation in the genome and this effect can be partially reversed by demethylation treatment. To confirm these results, an independent cell line was treated with 10 μM of AZA for 5 and 10 days. At the 10-day time-point, 61 of 155 allelically expressed loci (more than a two-fold difference in untreated) showed a 50% decrease in magnitude of AE upon treatment and no loci showed an opposite effect (that is, there was a 50% increase in AE upon treatment). Of the loci strongly affected by the treatment, 95% (58 of 61) showed consistent time dependency of treatment (at 5 days the magnitude change in AE was less marked). The directionality and time dependence of the treatment suggest that changes in AE were specific to AZA treatment. To further verify that demethylation was occurring, we incubated fragmented DNA with His-MBD2b, a methyl binding protein that has a high affinity for CpG methylated DNA. We then removed the non-tagged DNA, leaving only methylated fragments. Comparing the signal intensities (XY raw signals from 1M Illumina BeadChip) in DNA between the treated and untreated samples after the methyl binding protein affinity assay shows that, for sites where XY raw signal significantly differs (> 1 SD difference) between treated and untreated samples, the direction of effect is predominantly towards a decrease of signal intensities in treated cells, suggesting that AZA treatment did in fact reduce global methylation in LCLs.

**Table 4:** Genes affected by AZA treatment

			19099		19141			
						
Gene	Transcript	Location	Untreated	Treated	Untreated	Treated	IBD	Mapped to polymorphism
*PCTK3*	NM_212502	chr1:203742262-203768466	2.54	1.42	2.74	1.24	Yes	
*CR1L*	NM_175710	chr1:205886352-205961039	2.21	1.38	2.20	1.49	Yes	
*KCNK1*	NM_002245	chr1:231822688-231871795	1.78	1.11	4.69	2.59	NI	Yes
-		chr4:79778447-79803457	2.47	1.78	2.1	1.27	NA	Yes
-		chr5:173100613-173139917	2.33	1.27	2.08	1.15	Yes	
-		chr5:9599989-9600708	3.02	1.58	1.47	1.06	Yes	
-		chr6:139658229-139733915	3.16	2.36	3.53	2.53	Yes	Yes
-		chr6:80016628-80042343	3.16	2.36	2.71	1.25	NA	
*CALN1*	NM_001017440	chr7:71159735-71207121	1.51	1.05	5.76	1.76	NA	
-		chr11:3036678-3063235	4.64	2.94	4.63	3.18	Yes	Yes
*SYT9*	NM_175733	chr11:7376868-7440901	2.09	1.31	3.48	2.15	Yes	Yes
*VWA5A*	NM_001130142	chr11:123521934-123522703	2.02	1.37	2.12	1.37	Yes	
*P2RX7*	NM_002562	chr12:120055848-120087505	2.23	1.08	1.81	1.24	Yes	
*COL4A2*	NM_001846	chr13:109958305-109963202	3.25	1.5	2.58	1.84	Yes	
*PRKCH*	NM_006255	chr14:60959560-61030659	2.17	1.64	1.92	1.43	Yes	
*DNAJA4*	NM_00130182	chr15:76345564-76360674	2.15	1.58	8.56	5.77	Yes	
-		chr15:94684325-94711444	3.11	2.13	5.45	3.28	Yes	
*GAS7*	NM_001130831	chr17:10022884-10022981	4.24	2.21	2.13	1.44	Yes	Yes
-		chr17:14190861-14192673	2.37	1.68	2.34	1.60	NI	Yes
*C20orf194*	NM_001009984	chr20:3179134-3334482	3.47	2.08	1.92	1.45	Yes	
-		chr20:46050433-46119516	2.22	1.54	2.48	1.65	NA	
-		chr20:46358273-46404570	2.06	1.27	3.15	1.59	NA	Yes
*GNAS*	NR_002875	chr20:56848505-56882141	6.46	4.14	3.62	2.06	Yes	
*TMPRSS3*	NM_024022	chr21:42665938-42688945	1.47	1.04	4.19	1.43	Yes	
-		chr22:28762403-28805154	1.43	1.03	4.25	2.23	Yes	
*OSBP2*	NM_030758	chr22:29598129-29633708	2.41	1.84	2.86	1.71	NI	

IBD, identical-by-descent; NA, not available; NI, not informative.

**Table 5:** Genes not affected by treatment

			19099		19141			
						
Gene	Transcript	Location	Untreated	Treated	Untreated	Treated	IBD	Mapped to polymorphism
*MARK1*	NM_018650	chr1:218867493-218900613			6.38	6.26	Yes	
*DISC1*	NM_001012957	chr1:229837731-230086433			4.64	4.85	Yes	Yes
*CYP27A1*	NM_000784	chr2:219372907-219379842	3.84	2.54	1.25	1.62	NI	
*THNSL2*	NM_018271	chr2:88252911-88265923	2.43	3.37			Yes	Yes
*PTPRG*	NM_002841	chr3:62165281-62250653	4.02	3.09	3.65	5.10	NI	
*UPK1B*	NM_006952	chr3:120375223-120399317	4.12	3.68	1.23	1.33	NI	
*FAM53A*	NM_001013622	chr4:1654935-1655009	1.93	2.04	2.30	2.13	Yes	
*EVC*	NM_153717	chr4:5767823-5801057	1.68	1.78	3.47	3.11	Yes	
-		chr4:6698225-6722860			3.9	3.99	Yes	Yes
-		chr4:107011829-107032181			4.34	4.27	NI	
-		chr4:142529192-142768065	5.11	5.47			Yes	Yes
*ANKH*	NM_054027	chr5:14801236-14922709	1.42	1.35	7.59	8.63	Yes	Yes
-		chr5:82347320-82386566			2.1	2.23	Yes	Yes
-		chr6:654765-656792	3.18	3.13			NA	
*MOXD1*	NM_015529	chr6:132659162-132759924	2.02	2.08	3.07	3.03	Yes	Yes
-		chr8:511306-580861	3.54	2.76	2.33	2.79	Yes	Yes
-		chr9:5296824-5301171			3.83	3.91	Yes	
*DEC1*	NM_017418	chr9:117025707-117204395	2.22	2.18			Yes	Yes
*DIP2C*	NM_014974	chr10:363048-477973	3.56	2.48	1.25	1.73	Yes	
*FRMD4A*	NM_018027	chr10:13817200-14106528			3.93	4.13	NA	
-		chr11:6879025-6898447	2.81	2.20	1.63	2.15	Yes	Yes
-		chr11:70187425-70240934	2.73	2.13	1.52	1.83	NI	
-		chr13:18766583-18804422	3.50	3.03	3.52	4.30	Yes	Yes
*WDR51B*	NM_172240	chr12:88415605-88431297	2.18	2.27	2.66	2.62	Yes	Yes
-		chr14:24047434-24096337	2.44	2.01	4.27	5.26	NI	
*PAX9*	NM_006194	chr14:36198687-36216226	1.98	1.51	2.06	2.63	Yes	
-		chr14:69730226-69746414			5.76	5.81	NA	
*DPF3*	NM_012074	chr14:72343297-72429399	1.83	2.39	3.30	2.29	Yes	
*WARS*	NM_173701	chr14:99906106-99911812	2.49	2.41			Yes	Yes
-		chr15:22775434-22933834	11.24	9.95	7.06	8.26	Yes	
-		chr15:28921438-28971039	11.73	7.79	2.13	2.70	Yes	Yes
*SV2B*	NM_014848	chr15:89614733-89637888	2.88	2.74			NI	
-		chr16:53974720-54069307	5.38	2.88	1.10	1.64	Yes	Yes
-		chr16:83152950-83155553			3.52	3.6	NA	
*SLC13A5*	NM_177550	chr17:6531791-6555012			3.75	3.89	NA	
-		chr17:34566422-34580691	2.65	2.28	1.56	1.78	NA	
*PITPNC1*	NM_181671	chr17:63031387-63046267	3.08	2.46	1.59	1.83	Yes	
*DSC3*	NM_024423	chr18:26824546-26875293	1.88	2.21	4.83	3.67	Yes	Yes
*KATNAL2*	NM_031303	chr18:42780796-42812910	1.97	1.83	2.41	2.60	NI	
-		chr19:40008284-40033757	6.05	5.53	4.29	4.95	NI	
*SIGLEC5*	NM_003830	chr19:56807457-56823545	1.45	1.24	3.84	5.51	NI	
-		chr19:58776466-58798723	3.02	3.32			Yes	
-		chr22:22567862-22619365	1.12	1.03	8.09	10.02	Yes	Yes
*LDOC1L*	NM_032287	chr22:43268050-43270537	1.46	1.75	3.74	2.97	Yes	

IBD, identical-by-descent; NA, not available; NI, not informative.

**Table 6:** Allelic expression observed in identical-by-descent siblings

Condition	Number of loci	Concordant AE in independent IBD pairs	Discordant AE in independent IBD pairs
AE altered by AZA	26	32	14
AE not altered by AZA	48	67	7

AE, allelic expression; AZA, 5-azadeoxycytidine; IBD, identical-by-descent.

## Discussion

Our work demonstrates that many allelic expression events previously suggested to be caused by imprinting failed to validate in two human cell types, which allowed the detection of 59% of imprinted genes with stronger *a priori *evidence of parental expression bias and only 8% of imprinted genes with conflicting evidence of parental expression bias. These numbers suggest that caution is needed when experimentally assessing imprinting in the human genome. We note that while the transcriptome coverage is high (approximately 50% of RefSeq genes per tissue) using our methods, a limitation to the allelic expression mapping using primary transcripts is non-strand specificity; therefore, if antisense imprinting or imprinting of intragenic transcripts is common, we would underestimate the prevalence of imprinting. On the other hand, assessment of not commonly analyzed unannotated regions revealed few additional targets with potential imprinting. In addition to unannotated regions, our study included five-fold higher coverage for annotated genes than a previous allele-specific expression study [9] carried out in cells of lymphoid origin. Consequently, the coverage for validated imprinted genes was over five-fold higher for the LCLs in our study. Pollard *et al*. [9] assayed AE in 2,625 genes and only three of these were previously known to be imprinted.

In summary, we validated 20 genes out of the 41 genes we were able to assess for imprinting. Six genes were found imprinted in both LCLs and fibroblasts (*SNURF*, *IPW*, *ZNF597*, *ZNF331*, *GNAS*/*GNASAS *and *L3MBTL*). Most of the validated genes were found to be tissue-specific: *SGCE *and *KCNQ1 *were imprinted only in the LCLs while the other genes were imprinted only in the fibroblasts. Interestingly, 90% of the previously identified imprinted genes (18 of 20) validated in this study were imprinted in the primary fibroblasts as opposed to only 40% for the immortalized LCLs (8 of 20). For five of these genes we also found that the AE observed in the LCLs is mediated by heritable rather than epigenetic mechanisms (*PRIM2*, *CPA4*, *DLGAP2*, *ZNF215 *and *GABRG3*). Given the fact that *CPA4 *is found to be heritable in LCLs but imprinted in fibroblasts, further study of the two cell lines could help identify some of the factors involved in the mechanism of imprinting. Interestingly, another study found that *CPA4 *was imprinted in many fetal tissues but not in the fetal brain using pyrosequencing [38].

Several of the genes that were previously reported as imprinted (with consistent parent-of-origin transmission) were not confirmed in our study. In line with the literature, many of these are thought to be tissue-specific. For example, the gene *KCNK9 *is clearly imprinted but it is only highly expressed in the central nervous system and the cerebellum [39] and, as expected, shows no imprinting in LCLs and fibroblasts. The same thing can be said for the genes *PHLDA2 *and *OSBPL5*, which are imprinted in the placenta [40,41], and the genes *UBE3A *and *GRB10*, which are imprinted in the brain [42,43]. Based on the fact that we were able to validate 59% of the genes as having consistent parent-of-origin transmission compared to 8% validated as not having consistent parent-of-origin transmission, genes with inconsistent parent-of-origin transmission are more likely to be false positives.

Our data show conclusive evidence of imprinting for a few additional RefSeq genes (*NAT15 *and *SGK2*) as well as for three genes previously found imprinted in mice but not validated in humans (*ZDBF2*, *RTL1 *and *MEG8*) (Table 2). The *NAT15 *and *SGK2 *genes both lie adjacent to previously confirmed imprinted genes: *ZNF597 *and *L3MBTL*, respectively.

Our genome-wide analysis of unannotated regions revealed evidence of imprinting for four additional regions (Figure 2), all of which were identified in the fibroblasts. Three of these regions span multiple genes. In addition, we discovered four new genes with moderate imprinting (*TRAPPC9*, *ADAM23*, *CHD7 *and *TTPA*), all of which showed paternal expression. The observation of partial imprinting for *TRAPPC9 *is notable and should be studied in brain since this gene has recently been shown to be mutated in autosomal recessive mental retardation [44-46]. Consequently, if imprinting or partial imprinting can be replicated in human brain, paternally transmitted loss-of-function mutations could be enriched among individuals with intellectual disability.

This is the first genome-wide survey of imprinting using human primary cells. The use of human fibroblasts to uncover new imprinted genes and regions and to validate known imprinted genes was more efficient than the use of LCLs. Putatively, the epigenetic alterations upon immortalization and prolonged cell culture observed earlier [47] in LCLs can disrupt imprinted gene expression. To further study the true extent of imprinting, tissue-dependent expression of primary cells retrievable from blood (distinct cellular lineages compared to fibroblasts) should be pursued [48]. The overall coverage of suggested and established imprinted genes should represent adequate tissue sampling. We note that our ability to observe imprinting in approximately 50% of known imprinted genes in the current study is not substantially lower than that reported by Gregg *et al*. [18] when studying multiple regions in developing mouse brain, where 47 of 72 of known and measured imprinted genes showed parent-of-origin-dependent expression. In contrast to this latter study and despite our high transcriptome coverage, we did not find widespread evidence of unknown classically imprinted genes or even partial imprinting in annotated or unannotated regions. One potential explanation for the difference in uncovering novel imprinted genes between our study and the study by Gregg *et al*. is that we required consistent parent-of-origin-dependent expression across a genomic region (three independent SNPs required) and most of the novel imprinting candidates observed in mice did not show consistent evidence across a transcriptional unit [18].

While the LCLs provide a less powerful cell system to study imprinting compared to primary fibroblasts, they offer the possibility to look for determinants of non-heritable allelic expression since the cells have reduced mosaicism and show an excess of extreme allelic expression compared to primary cells [32]. Gimelbrant and colleagues [25] have shown in individually derived LCL clones that the extent of RME could be substantial, but the mechanisms involved in random allelic silencing have not been previously pursued on a genome-wide scale. Here we show directly that reversible methylation is one of the mechanisms involved in RME using a demethylating agent in two different sets of samples. We also suggest that the mechanisms underlying transient methylation-mediated allelic silencing are not primarily involved in imprinting or heritable allelic expression since such loci were relatively underrepresented among loci showing allelic expression changes upon demethylation.

## Conclusions

In our comprehensive genome-wide search for imprinting and non-heritable allelic expression in human we found relatively few new imprinted genes, at least in LCLs and fibroblasts. Our results also suggest that the false-positive rate among suggested imprinted genes without direct parent-of-origin expression is high. This is likely, in part, due to the high prevalence of heritable allelic expression we observed in many candidate regions in our survey as well as technical issues in measuring allelic expression in human samples using single-point assessment. The existence of widespread parent-of-origin-dependent allelic expression observed recently in mouse studies [18] was not directly addressed in our assessment as we required multiple consistent measurements across transcripts. Overall, this could point to less than 100 classically imprinted genes (accounting for some tissue specificity) in the human genome. To extend the human catalogue where imprinting is directly observed as we show here, we suggest that other primary cells retrievable by non-invasive means (allowing analyses in pedigrees) will likely be needed.

## Materials and methods

### Imprinted gene search

Genes were selected from the imprinting catalogue maintained at the Catalogue of Parent of Origin Effects (University of Otago). Imprinted genes were categorized as having either consistent (44 genes selected) or inconsistent parent-of-origin transmission (13 genes selected).

### Samples and cell culture

For the lymphoblast samples, a three-generation pedigree of Caucasian origin (CEPH family 1420) [32] along with newly generated AE profiles in a Caucasian (1463) as well as a Yoruban (Y117) parent-offspring trio were used. In addition, nine independent parent-offspring fibroblast trios to confirm parental influence in AE were utilized. Seven of the loci showing parent-of-origin effects in LCLs had previously been validated by independent AE measurements in a second pedigree (1444) [32]. All LCLs were obtained from Coriell (Camden, NJ, USA) and fibroblast cell lines were also obtained from Coriell and the McGill Cellbank (Montreal, QC, Canada). Details of the cell lines used can be found in Table S4 in Additional file 1. This study was approved by the local ethics committee (McGill University IRB).

The HapMap immortalized LCLs were grown in T75 flasks in 1X RPMI 1640 Media (Invitrogen, Burlington, ON, Canada), with 2 mM L-glutamine, 15% fetal bovine serum and 1% (penicillin/streptomycin) at 37°C with 5% CO_2_. Fibroblasts primary cell lines were grown in medium containing a-MEM (SigmaAldrich, Oakville, ON, Canada) supplemented with 2 mmol/l L-glutamine, 100 U/ml penicillin, 100 mg/ml streptomycin, and 10% fetal bovine serum (SigmaAldrich) at 37°C with 5% CO_2_. At 70 to 80% confluence, the cells were harvested and stored at -70°C until RNA and DNA extraction.

### RNA and DNA extraction and cDNA synthesis

Total RNA was extracted from cell lysates resuspended in 600 ml RLT lysis buffer using the RNeasy Mini Kit (Qiagen, Ontario, Canada). High RNA quality was confirmed for all samples using the Agilent 2100 Bio-Analyzer (Agilent Technologies, Mississauga, ON, Canada) and the concentrations were determined using Nanodrop ND-1000 (NanoDrop Technologies, Wilmington, DE, USA). A cDNA synthesis protocol was applied on the heteronuclear DNA, and allowed the measurement of unspliced primary transcripts. Approximately 150 mg of total RNA was isolated, treated with 6 U DNase I and poly(A). The RNA was then enriched using the MicroPoly(A)Purist protocol (Ambion Inc., Streetsville, ON, Canada). The first- and second-strand cDNA synthesis was carried out on 1 μg poly(A)-enriched RNA using random hexamers and second strand cDNA synthesis was performed using the Superscript Double-Stranded cDNA Synthesis Kit (Invitrogen). DNA was extracted from cell lysates resuspended in 200 ml phosphate-buffered saline using the GenElute DNA Miniprep Kit (SigmaAldrich). Concentrations were determined using the Quant-iT PicoGreen kit (Invitrogen).

### Allelic expression analysis on Human1M or Human1M-Duo beadchips

Approximately 200 ng of genomic DNA and a 50 to 300 ng double-stranded cDNA sample were used for the parallel genotyping and AE analysis on the Illumina Infinium Human1M or Human1M-Duo SNP bead microarray as previously described [32]. The parallel assessment of gDNA and cDNA heterozygote ratios was carried out essentially as described earlier [32], but signal intensity normalization at heterozygous sites followed a slightly modified approach. For the AE analysis, we utilized the Xraw and Yraw signal intensities and since the variances in the two channels were not the same (that is, it is a function of total intensity from both channels), a normalization of the variation was performed to allow comparison between gDNA and cDNA allele ratios. In this study, only the β ratio was normalized (Xraw/(Xraw + Yraw)) from heterozygous SNPs with a total intensity (Xraw + Yraw) higher than the threshold value of 1,000. The scatter plot of the β ratio against the logarithm 10 scaled total intensity fits well with polynomial regression model (quadratic regression model). This model shows a better fit than the linear regression model that we employed earlier for normalization [32], which works well in higher intensity parts but poor in lower intensity parts in many samples. The normalization process can be briefly summarized into the following steps: step 1, the β ratio is calculated along with total intensity in log10 scale for all heterozygous SNPs; step 2, all data points with greater than 1,000 in total intensity are divided into 50 intensity bins; step 3, a fitted curve from the median β ratio in each bin is computed using a polynomial regression model (quadratic regression) y = b1x + b2 × 2 + a, where y is the expected β ratio from the curve and × is the log10 scaled total intensity; step 4, from the fitted curve, the expected β ratio based on total intensity is calculated; step 5, the final normalized β ratio equals (βobs - βexpected + 0.5). Following normalization, all median β ratio values in all intensity bins should be close, if not equal, to 0.5. Phasing of the genotypes in the trios were done using Beagle [49] and in the three-generation pedigree by Merlin [50].

### Validation of imprinted genes and genomic regions

Genes were considered to be imprinted if they had extreme AE with an average of more than 2.9-fold difference (1 SD calculated from genome-wide population data) between the two alleles as well as observation of transmission of AE that is consistent with paternal or maternal imprinting.

For novel imprinted genes and genomic regions, at least three consecutives SNPs needed to show extreme AE (> 2.9-fold) for them to be included in the analysis. For partial imprinted genes and regions, AE levels were required to fall within 2- to 2.9-fold average difference among all informative heterozygotes. Windows were calculated using a previously published method [32].

Validation of the Illumina Array was performed by measuring AE with normalized Sanger sequencing in LCL and fibroblast samples heterozygous for specific SNPs. Paired genomic DNA and cDNA from the samples were amplified for a specific SNP, verified by agarose gel electrophoresis and sequenced with ABI Big Dye chemistry and capillary electrophoresis on an ABI 3730 sequencer (Applied Biosystems, Foster City, CA, USA). The relative allelic expression levels for each SNP were assessed with the Peak-Picker software [34] and allele ratios below 0.1 or above 10 were assigned a value of 0.1 or 10, respectively, as they represent monoallelic expression (indistinguishable from homozygous sites). Similarly, estimated allele ratios below 0.1 or above 10 from the Illumina 1M assay were also assigned these values as they do not significantly differ from the homozygote ratios in BeadChip genotyping.

### Heritability

Variants showing extreme AE were assessed for heritability of the AE using population mapping data for the same cell type and for transmission compatible with Mendelian inheritance in the pedigrees.

### Demethylation treatment

Two lymphoblast cell lines (19099 and 19141) were treated with three concentrations (1, 5 and 10 μM) of the demethylating drug AZA every 24 hours for 3 days. For these treatment groups, the viability was 73%, 69% and 68%, respectively. We chose to use a concentration of 5 μM for treatment studies in these two cell lines. A third LCL (12892) was treated with 10 M AZA for 5 and 10 days. Total RNA was collected and prepared for genome-wide AE analysis at each time point and in untreated controls as described above.

To confirm demethylation, we also collected DNA in untreated and treated states from 12892.We combined the 5- and 10-day treatment groups as there was insufficient DNA for the 10-day group alone. We fragmented 10 μg of DNA by mixing it with TE buffer and nebulization buffer placed in a nebulizer cup. Forty-five psi of nitrogen was passed through the nebulizer cup for 1 minute in order to fragment the DNA. The DNA was then purified using a Qiagen MiniElute PCR Purification kit (Qiagen). Qiagen's buffer PBI was added and it was passed through a spin column, then PE was passed through the column, then buffer EB to elute the DNA. Next was an AMPure bead purification step in order to isolate the appropriate size fragments required (over 1,000 bp). Buffer EB and AMPure beads were added to the DNA. Then the beads were collected using a magnetic particle concentrator, washed with ethanol and finally the DNA was eluted from the beads using buffer EB.

A methyl collector version B1 (Active Motif, Carlsbad, CA, USA) was used to isolate methylated CpG islands from fragmented genomic DNA according to the manufacturer's protocol in order to verify demethylation of the DNA upon AZA treatment. In the first step, 1 μg of DNA was mixed with His-MBD2b protein, along with the binding buffer provided and magnetic beads to capture the protein-DNA complex. Next, the beads were collected by the magnetic particle concentrator, the beads were washed with more binding buffer, and finally the beads were collected again and the supernatant discarded. Lastly, the methylated fragments were recovered by incubating the solution with the provided elution buffer.

### Transmission analyses

Transmission patterns from parent to offspring for AE loci were assessed in the above-mentioned families (two LCL CEPH families, one LCL Caucasian trio, one LCL Yoruba trio and nine fibroblasts trios). Patterns consistent with imprinting were observed when the overexpressed allele always came from the same parent regardless of which allele was associated with overexpression in the parent.

### Population mapping data

Mapping of heritable AE traits in CEU LCLs has been previously reported by us [32]. For the fibroblasts, a similar approach for population mapping was employed, using 64 unrelated primary fibroblasts from parent-offspring trios (most of the children were only analyzed for genotypes in DNA in order to phase the parental allelic expression data). These parental samples were phenotypically normal donors of Caucasian origin. The genome-wide mapping of AE in primary fibroblasts will be reported separately.

## Abbreviations

AE: allelic expression; AZA: 5-azadeoxycytidine; IBD: identical-by-descent; LCL, lymphoblast cell line; RME: random monoallelic expression; SD: standard deviation; SNP: single-nucleotide polymorphism.

## Competing interests

The authors declare that they have no competing interests.

## Authors' contributions

TP conceived research; DKP, LM, DJV and TP designed experiments; LM, VK, KCLL, AM and DJV conducted experiments; BG, DKP, and TP designed computational and analytical methods; BG, LM, DJV, KLG and TP analyzed data; TP, LM and DJV drafted the manuscript and all authors contributed to final manuscript writing and its revision.

## Supplementary Material

Additional file 1 **Tables S1, S2, S3, and S4**. Tables of loci not imprinted, uninformative loci or of loci used in the validation as well as a description of LCL and fibroblast samples. Click here for file

Additional file 2 **Figure S1**. Figure demonstrating the correlation of AE between normalized Sanger sequencing and the Illumina array. Click here for file

Additional file 3 **Tables S5 and S6**. Candidate windows in LCLs and fibroblasts showing high allelic expression. Click here for file

Additional file 4 **Figure S2**. Figure demonstrating four loci showing imprinted expression. Click here for file
