# Supplementary material for: Genome-wide assessment of imprinted expression in human cells
Source: Genome Biol. 2011 Mar 21;12(3):R25. doi: 10.1186/gb-2011-12-3-r25 (PMC3129675; doi:10.1186/gb-2011-12-3-r25)
Supplement: Additional file 1 — Tables S1, S2, S3, and S4. Tables of loci not imprinted, uninformative loci or of loci used in the validation as well as a description of LCL and fibroblast samples. [file gb-2011-12-3-r25-S1.DOC]

**Table S1: Evidence against imprinting in LCL and/or FB**

I: Imprinting, ND: not determined, NA: not available, P: paternal, M:maternal, PD: provisional data, CD: conflicting data, NO: No ortholog, LCL: lymphoblast, FB: fibroblast

| **Location** | **Gene** | **Human** | **Mouse** | **Expressed**  **allele** | **LCL** | **FB** |
| --- | --- | --- | --- | --- | --- | --- |
| 1p36 | *TP73* | I | ND | M | no | no |
| 6p11 | *PRIM2* | I | I | M | no | no |
| 7p12 | *GRB10* | I | I | P/M | no | NA |
| 7q21 | *CALCR* | PD | I | M | no | NA |
| 8p23 | *DLGAP2* | I | ND | P | no | no |
| 8q24 | *KCNK9* | I | I | M | no | no |
| 10q26 | *INPP5F_V2* | I | I | P | no | no |
| 11p15 | *SLC22A1LS* | PD | NO | M | no | NA |
|  | *SLC22A18* | I | I | M | no | no |
|  | *PHLDA2* | I | I | M | NA | no |
|  | *OSBPL5* | I | I | M | NA | no |
|  | *ZNF215* | PD | NO | M | no | no |
| 11q23 | *SDHD* | CD | ND | P | no | NA |
| 13q14 | *HTR2A* | CD | I | M | no | no |
| 15q11 | *UBE3A* | I | I | M | no | no |
| 15q12 | *ATP10A* | I | CD | M | no | no |
|  | *GABRB3* | CD | ND | P | NA | no |
|  | *GABRA5* | CD | ND | P | NA | no |
|  | *GABRG3* | CD | ND | P | no | NA |
| 19q13 | *PEG3* | I | I | P | no | NA |

**Table S2: Uninformative loci in LCL and FB**

| **Location** | **Gene** | **Human** | **Mouse** | **Expressed allele** | **LCL** | **FB** |
| --- | --- | --- | --- | --- | --- | --- |
| 1p31 | *DIRAS3* | PD | NO | P | NA | NA |
| 6q24 | *HYMAI* | I | NR | P | NA | NA |
| 2p12 | *LRRTM1* | I | NR |  | NA | NA |
| 7q21 | *PON1* | PD | NI | P | NA | NA |
|  | *DLX5* | I | NI | M | NA | NI |
| 11p15 | *IGF2* | I | I | P | NA | NI |
|  | *INS* | I | I | P | NA | NA |
|  | *ASCL2* | CD | I | M | NA | NA |
|  | *TRPM5* | PD | NI | P | NA | NA |
|  | *KCNQ1DN* | I | NO | M | NA | NA |
|  | *CDKN1C* | I | I | M | NA | NA |
| 11p13 | *WT1-Alt transcript* | I | NR | P | NA | NA |
| 18q21 | *TCEB3C* | I | NO | M | NA | NA |
| 19q13 | *ITUP1* | I | NO | P | NA | NA |
| 20q11 | *NNAT* | I | I | P | NA | NA |
|  | *PSIMCT-1* | I | I | P | NA | NA |

I: imprinting, P: paternal, M: maternal, NA: not available, NI: not informative, LCL: lymphoblast, FB: fibroblast, PD: provisional data, CD: conflicting data, NO: no ortholog, NR: not reported.

**Table S3:** Validation of Illumina Array

| **Gene** | **SNP** | **# of LCLs** | **# of FBs** |
| --- | --- | --- | --- |
| *GNAS* | rs6070638 |  | 3 |
| *KCNQ1* | rs10832514 |  | 4 |
| *L3MBTL* | rs2071970 | 1 |  |
|  | rs6030939 |  | 1 |
| *MEG8* | rs2180386 |  | 3 |
| *PEG10* | rs13073 | 3 | 3 |
|  | rs13226637 | 2 | 3 |
| *PTPRB* | rs630608 |  | 6 |
| *RTL1* | rs3825569 |  | 4 |
| *SNRPN* | rs705 | 3 | 1 |
| *ZDBF2* | rs10932150 |  | 10 |
|  | rs12694049 |  | 8 |
| *ZNF597* | rs37831 | 2 |  |
| Intergenic region | rs3809404 |  | 3 |

**Table S4: Sample description**

| **Cell Type** | **Trio #** | **Coriell (GM)/ McGill (WG) #** | **Disease** | **Sex** | **Age (yr)** | **Origin** | **Affected** | **Relation** |
| --- | --- | --- | --- | --- | --- | --- | --- | --- |
| FB | 1 | GM00519 | Mucopolysaccharidosis type VI | F | 4 | CEU | Yes | Daughter |
|  | 1 | GM00520 | Mucopolysaccharidosis type VI | M | 42 | CEU | No | Father |
|  | 1 | GM00935 | Mucopolysaccharidosis type VI | F | 40 | CEU | No | Mother |
|  | 2 | WG1656 | Transcobalamin II deficiency | M | 4 | CEU | Yes | Son |
|  | 2 | WG1657 | Transcobalamin II deficiency | M |  | CEU | No | Father |
|  | 2 | WG1658 | Transcobalamin II deficiency | F |  | CEU | No | Mother |
|  | 3 | WG1226 | CblC | M | 0.25 | CEU | Yes | Son |
|  | 3 | WG1282 | CblC | M |  | CEU | No | Father |
|  | 3 | WG1283 | CblC | F |  | CEU | No | Mother |
|  | 4 | WG1084 | MTHFR deficiency |  |  | CEU | Yes | Child |
|  | 4 | WG2844 | MTHFR deficiency | M |  | CEU | No | Father |
|  | 4 | WG2843 | MTHFR deficiency | F |  | CEU | No | Mother |
|  | 5 | GM00289 | Fucosidosis | M | 31 | CEU | No | Father |
|  | 5 | GM00290 | Fucosidosis | F | 29 | CEU | No | Mother |
|  | 5 | GM00292 | Fucosidosis | M | 4 | CEU | Yes | Son |
|  | 6 | GM01391 | Hurler syndrome | F | 0.75 | CEU | Yes | Daughter |
|  | 6 | GM01392 | Hurler syndrome | F | 24 | CEU | No | Mother |
|  | 6 | GM01393 | Hurler syndrome | M | 24 | CEU | No | Father |
|  | 7 | GM02314 | Farber lipogranulomatosis | F | 6 | CEU | Yes | Daughter |
|  | 7 | GM02316 | Farber lipogranulomatosis | M | 37 | CEU | No | Father |
|  | 7 | GM02317 | Farber lipogranulomatosis | F | 37 | CEU | No | Mother |
|  | 8 | GM02455 | Mucopolysaccharidosis type IVB | F | 6 | CEU | Yes | Daughter |
|  | 8 | GM02456 | Mucopolysaccharidosis type IVB | M | 30 | CEU | No | Father |
|  | 8 | GM02555 | Mucopolysaccharidosis type IVB | F | 30 | CEU | No | Mother |
|  | 9 | GM02639 | Diabetes mellitus, juvenile-onset insulin-dependent; IDDM | M | 19 | CEU | No | Son |
|  | 9 | GM02640 | Diabetes mellitus, juvenile-onset insulin-dependent; IDDM | M | 41 | CEU | No | Father |
|  | 9 | GM02641 | Diabetes mellitus, juvenile-onset insulin-dependent; IDDM | F | 41 | CEU | No | Mother |
| LCL | 1463 | GM12891 |  | M |  | CEU |  | Father |
|  | 1463 | GM12892 |  | F |  | CEU |  | Mother |
|  | 1463 | GM12878 |  | F |  | CEU |  | Daughter |
|  | Y117 | GM19239 |  | M |  | YRI |  | Father |
|  | Y117 | GM19238 |  | F |  | YRI |  | Mother |
|  | Y117 | GM19240 |  | F |  | YRI |  | Daughter |
