# Supplementary figures and images for: Genome-wide assessment of imprinted expression in human cells
Source: Genome Biol. 2011 Mar 21;12(3):R25. doi: 10.1186/gb-2011-12-3-r25 (PMC3129675; doi:10.1186/gb-2011-12-3-r25)

## Slide 1
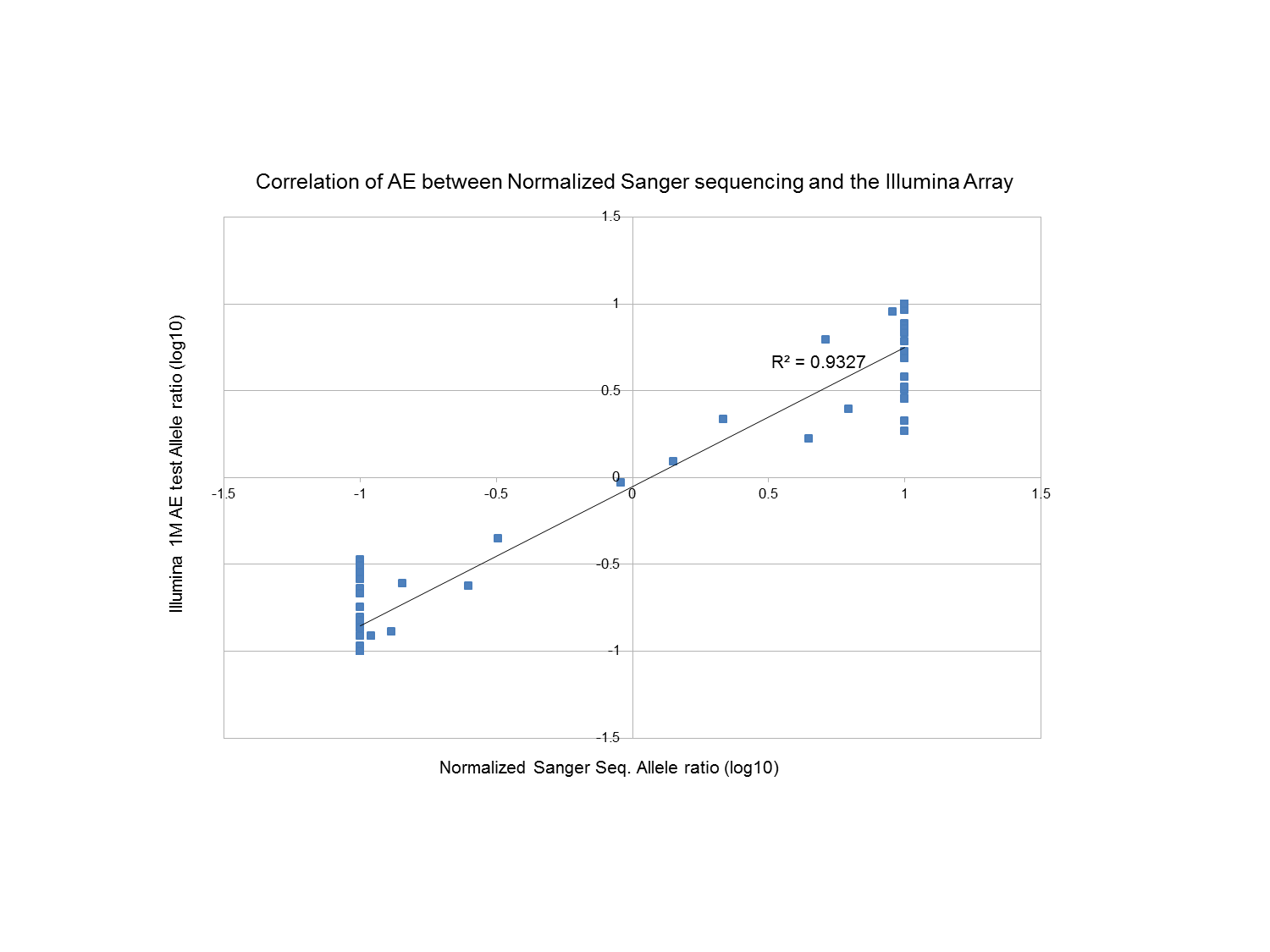

Supplement: Additional file 2 — Figure S1. Figure demonstrating the correlation of AE between normalized Sanger sequencing and the Illumina array. [file gb-2011-12-3-r25-S2.PPT]
