# Supplementary material for: Genome-wide assessment of imprinted expression in human cells
Source: Genome Biol. 2011 Mar 21;12(3):R25. doi: 10.1186/gb-2011-12-3-r25 (PMC3129675; doi:10.1186/gb-2011-12-3-r25)
Supplement: Additional file 4 — Figure S2. Figure demonstrating four loci showing imprinted expression. [file gb-2011-12-3-r25-S4.PPT]

## Slide 1
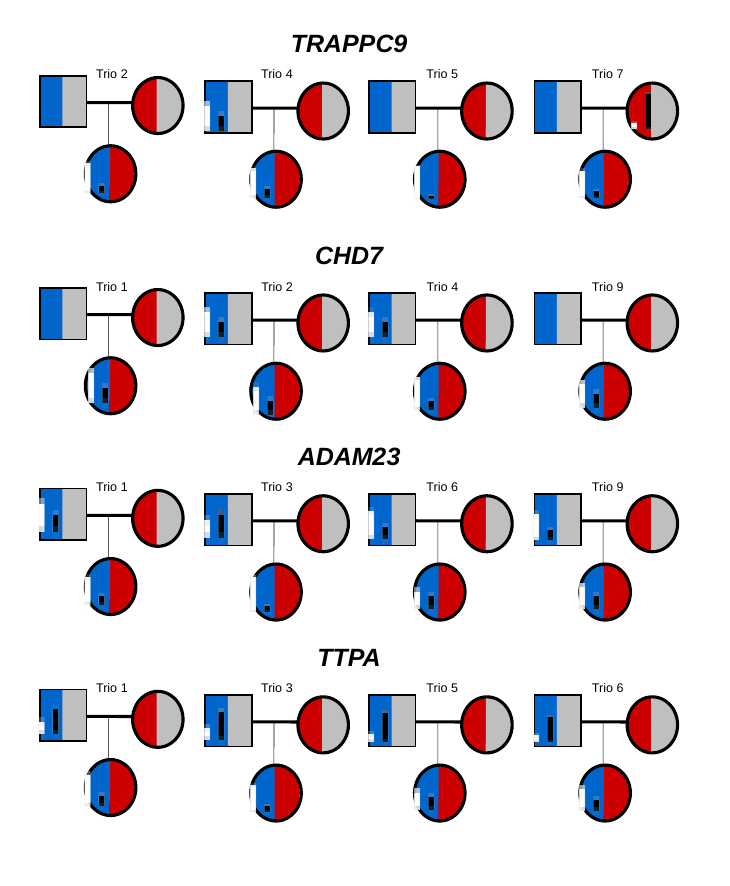

TRAPPC9
Trio 2
Trio 4
Trio 5
Trio 7
CHD7
Trio 1
Trio 2
Trio 4
Trio 9
ADAM23
Trio 1
Trio 3
Trio 6
Trio 9
TTPA
Trio 1
Trio 3
Trio 5
Trio 6
